# Rate-dependent C*a*^2+^ signalling underlying the force-frequency response in rat ventricular myocytes: a coupled electromechanical modeling study

**DOI:** 10.1186/1742-4682-10-54

**Published:** 2013-09-10

**Authors:** Abhilash Krishna, Miguel Valderrábano, Philip T Palade, John W Clark

**Affiliations:** 1 Department of Electrical and Computer Engineering, Rice University, Houston, Texas, USA; 2 Methodist Hospital Research Institute, Methodist DeBakey Heart & Vascular Center, Houston, Texas, USA; 3 Department of Pharmacology and Toxicology, University of Arkansas for Medical Sciences, Little Rock, Arkansas, USA

## Abstract

**Background:**

Rate-dependent effects on the *Ca*^2+^ sub-system in a rat ventricular myocyte are investigated. Here, we employ a deterministic mathematical model describing various *Ca*^2+^ signalling pathways under voltage clamp (VC) conditions, to better understand the important role of calmodulin (CaM) in modulating the key control variables *Ca*^2+^/calmodulin-dependent protein kinase-II (CaMKII), calcineurin (CaN), and cyclic adenosine monophosphate (cAMP) as they affect various intracellular targets. In particular, we study the frequency dependence of the peak force generated by the myofilaments, the force-frequency response (FFR).

**Methods:**

Our cell model incorporates frequency-dependent CaM-mediated spatially heterogenous interaction of CaMKII and CaN with their principal targets (dihydropyridine (DHPR) and ryanodine (RyR) receptors and the SERCA pump). It also accounts for the rate-dependent effects of phospholamban (PLB) on the SERCA pump; the rate-dependent role of cAMP in up-regulation of the L-type *Ca*^2+^ channel (*I*_*Ca*,*L*_); and the enhancement in SERCA pump activity via phosphorylation of PLB.

**Results:**

Our model reproduces positive peak FFR observed in rat ventricular myocytes during voltage-clamp studies both in the presence/absence of cAMP mediated *β*-adrenergic stimulation. This study provides quantitative insight into the rate-dependence of *Ca*^2+^-induced *Ca*^2+^-release (CICR) by investigating the frequency-dependence of the trigger current (*I*_*Ca*,*L*_) and RyR-release. It also highlights the relative role of the sodium-calcium exchanger (NCX) and the SERCA pump at higher frequencies, as well as the rate-dependence of sarcoplasmic reticulum (SR) *Ca*^2+^ content. A rigorous *Ca*^2+^ balance imposed on our investigation of these *Ca*^2+^ signalling pathways clarifies their individual roles. Here, we present a coupled electromechanical study emphasizing the rate-dependence of isometric force developed and also investigate the temperature-dependence of FFR.

**Conclusions:**

Our model provides mechanistic biophysically based explanations for the rate-dependence of CICR, generating useful and testable hypotheses. Although rat ventricular myocytes exhibit a positive peak FFR in the presence/absence of beta-adrenergic stimulation, they show a characteristic increase in the positive slope in FFR due to the presence of Norepinephrine or Isoproterenol. Our study identifies cAMP-mediated stimulation, and rate-dependent CaMKII-mediated up-regulation of *I*_*Ca*,*L*_ as the key mechanisms underlying the aforementioned positive FFR.

## Background

Cardiac muscle contraction is a result of a transient increase in intracellular *Ca*^2+^ concentration[ *Ca*^2+^] _*myo*_. Sarcolemmal (SL) membrane depolarization triggers *Ca*^2+^ influx via the dihydropyridine (DHP)-sensitive L-type *Ca*^2+^ channels. Following diffusion across a small sub-membrane dyadic space, this influx activates ryanodine receptors (RyRs) controlling ryanodine-sensitive *Ca*^2+^ release channels in the junctional portion of the sarcoplasmic reticulum (jSR). Fabiato and Fabiato [1] named the process calcium-induced calcium release (CICR). *Ca*^2+^ subsequently diffuses from the dyadic space into the myoplasm. Ultimately, intracellular *Ca*^2+^ concentration [ *Ca*^2+^] _*myo*_ is returned to resting levels by combination of: (a) *Ca*^2+^ buffering in the dyadic space and myoplasm; (b) sequestration of *Ca*^2+^ by sarcoplasmic/endoplasmic reticulum *Ca*^2+^-ATPase (SERCA)-type calcium pumps lining the longitudinal portion of the sarcoplasmic reticulum (LSR); and (c) *Ca*^2+^ extrusion from the myoplasm by *Na*^+^/ *Ca*^2+^ exchangers and *Ca*^2+^-ATPase pumps on the sarcolemmal membrane.

*Ca*^2+^ is an extremely important and highly versatile second messenger in cardiac cells, which plays a crucial role not only in excitation-contraction (E-C) coupling but also in excitation-transcription coupling [2]. Various inter-connected *Ca*^2+^ signalling pathways help preserve the integrity of the cellular *Ca*^2+^ system despite any changes in pacing frequency. Specifically, *Ca*^2+^ triggers the CaM-mediated rate-dependent effects of CaMKII and CaN on the characteristics of the apposed dihydropyridine (DHP) and ryanodine-sensitive *Ca*^2+^ channels in the dyad, whose interaction forms the basis for CICR. The proteins CaMKII and CaN not only influence the release mechanism but also affect the SERCA pump either directly or indirectly via phospholamban (PLB), thus modulating the *Ca*^2+^ uptake process [3-6]. It is also known that *β*-adrenergic stimulation of the cardiac cell, mediated by the second messenger cAMP, modulates the frequency dependence of the peak force generated by the myofilaments, the force-frequency response (FFR). These protein-mediated and second messenger pathways help maintain *Ca*^2+^ homeostasis over a wide range of stimulation frequencies.

Cardiac contractile function is closely coupled with heart rate (Bowditch effect [7]). Although positive [8], almost flat [9] and negative [10,11] peak FFR have been reported in the literature, it is clear from in-vitro studies involving stimulation in the physiological range of frequencies [12,13] that rat ventricle exhibits a positive peak FFR. The issue of force-frequency relationship requires broadened investigation into the various underlying cellular and molecular mechanisms. We propose that mathematical modeling would be a useful tool in helping to sort out this complex issue.

## Methods

**Figure 1:**
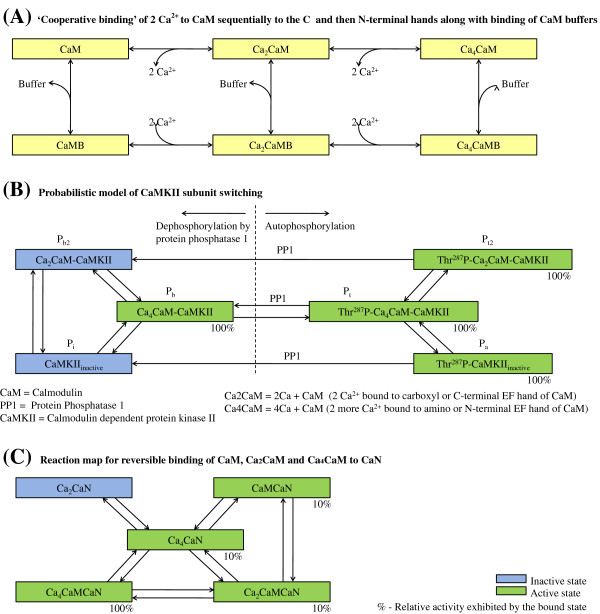
**CaM-CaMKII-CaN pathways.** Reaction Maps. **(A)** Cooperative binding of 2 *Ca*^2+^ to CaM sequentially to the C and then N-terminal hands along with binding of CaM buffers; **(B)** Probabilistic model of CaMKII subunit switching; **(C)** Reaction map for reversible binding of CaM, Ca _2_CaM and Ca _4_CaM to CaN.

All simulations and analysis were performed on a 2.8GHz Intel ^*Ⓡ*^ Core ^TM^2 Duo CPU-based computer using Microsoft Windows XP operating system. The sarcolemmal membrane charge balance equations, the *Ca*^2+^ material balance equations in the myoplasm and SR, and the force balance equations describing the model for myofilament contraction constitute a set of 93 ordinary differential equations (ODEs). A fixed-step Merson-modified Runge-Kutta 4th-order numerical integration scheme [14] was used to solve this set of 1st-order differential equations (ODE) describing the dynamic model. The free *Ca*^2+^ concentration in the dyad is governed by the time courses of the *Ca*^2+^ fluxes through *Ca*^2+^ transport systems, as well as by the time course of *Ca*^2+^ binding to *Ca*^2+^ buffers present in the junction [15]. Description of the spatio-temporal dynamics of calcium transients in the dyad triggered by *Ca*^2+^ stimulus (basis of CICR) requires calculation of the partial differential equations (PDE) of the whole reaction-diffusion system. Formation and dissipation of *Ca*^2+^ gradients around an open channel (DHP-sensitive and Ry-sensitive channels in the dyad) is assumed instantaneous as was validated for microsecond timescale and nanoscopic space by Naraghi and Neher [16]. Local *Ca*^2+^ concentration in the vicinity of open channels (located on opposing boundaries of the dyadic space) was calculated as the steady state gradient around a point source [17]. The *Ca*^2+^ concentration increments from individual channels at each point in space were assumed to be additive [16,18]. The software kernel follows the changes in the state of trigger and release channels together with variables like membrane voltage and spatial *Ca*^2+^ concentration to calculate the instantaneous rate constants and estimate the duration of transient events. Crank [19] discusses diffusion problems in a two-phase heterogeneous medium and shows that diffusion through a system of barriers (RyR feet structures in the dyadic cleft space) can be approximated by diffusion in the same region without barriers but with a reduced effective diffusion coefficient. We hence take this approach in modeling the *Ca*^2+^ diffusion by solving the 2-D Laplacian equation (Krishna et al. [15], Appendix A3, Eq. 140) in the DCU without explicitly accounting for local potential fields. More specifically, an explicit finite difference scheme was used to solve these Laplacian equations describing *Ca*^2+^-diffusion in the dyadic space analogous to the method detailed in Smith et al. [20]. Specifically, a radial symmetry is employed in solving the PDE in the dyadic volume allowing the solution to be computed in a rectangular cross-section discretized into a 20 by 20 cartesian grid. The spatial step size used in the r and z-direction (Figure 1B, Krishna et al. [15]) was 10 nm and 0.76 nm respectively (Table two, Krishna et al. [15]). The 20x20 grid size was used to obtain a stable numerical solution using the explicit finite difference scheme employed to solve the PDE. Obtaining an accurate description for *Ca*^2+^-diffusion in the dyadic space is vital to ensure adequate time delays associated with RyR release (z-direction) and *Ca*^2+^-diffusion into the cytosol (r-direction) which controls the rate of SR *Ca*^2+^-uptake via the SERCA pump. Both of these delays are important in ensuring robust luminal sensor mediated RyR refractory characteristics (described in Krishna et al. [15]). We use the method of lines (discretization in space) to solve the PDE. The full set of ODEs and finite difference equations are solved simultaneously to obtain the complete solution. Execution of a single cycle which translates to 200 ms at 5 Hz took 21 seconds with a time step of 1 *μ*s. A non-linear leastsquares method [21] was used for parameter estimation and data fitting. Results were visualized using Matlab (Mathworks, Natick, MA) and Origin (OriginLab Corp., Northampton, MA).

## Model development

Our objective was to develop a model of the rat ventricular cell under voltage clamp conditions, which includes the description of various *Ca*^2+^ signalling pathways in the dyadic space, the myoplasmic medium and the sarcoplasmic reticulum. Therefore, we start with a broad discussion of the key proteins involved in the *Ca*^2+^ signalling mechanism and continue with a progressively more detailed description of their influence on respective targets. It is important to note that all *Ca*^2+^ concentrations discussed in the model pertain to unbound *Ca*^2+^ unless specified. A detailed description of the membrane classification, channel and exchanger distribution as well as the various fluid compartments involved is given in Krishna et al. [15].

Calmodulin mediates the regulation of a variety of *Ca*^2+^-dependent signalling pathways in the heart involving CaMKII and CaN [2,22]. These protein-mediated interactions form the basis for a robust mechanism that enables a cell’s response to increased heart rate. CaMKII is reported to be responsive when targeted to *Ca*^2+^ release sites such as the dyadic cleft, and CaN is responsive to gradual changes in the lower-amplitude myoplasmic *Ca*^2+^ signals [2]. This heterogenous response is a result of the different affinities of CaMKII and CaN for CaM [22] and the non-uniform distribution of these proteins in the cell. Recent study [23] also attributes a key role in frequency-dependent acceleration of relaxation to activated-CaMKII in the cytosol. The model of the CaM-dependent *Ca*^2+^ signalling process (Figure 1), which includes a reaction map for cooperative binding of *Ca*^2+^ to CaM, the scheme for CaM buffering, probabilistic model of CaMKII subunit switching and the reaction map for reversible binding of CaM, Ca_2_CaM and Ca_4_CaM to CaN, is adopted from Saucerman et al. [2]. However, to reproduce relative local CaMKII and CaN activity, modifications were made to the rate constants for CaM buffering in the dyad (Table five, Krishna et al. [15]). Specifically, a limitation of the Saucerman and Bers model is that it is based on little available information regarding the operation of CaM buffers [24] at locations where CaM encounters very high *Ca*^2+^ concentrations (compared with the myoplasm).

**Figure 2:**
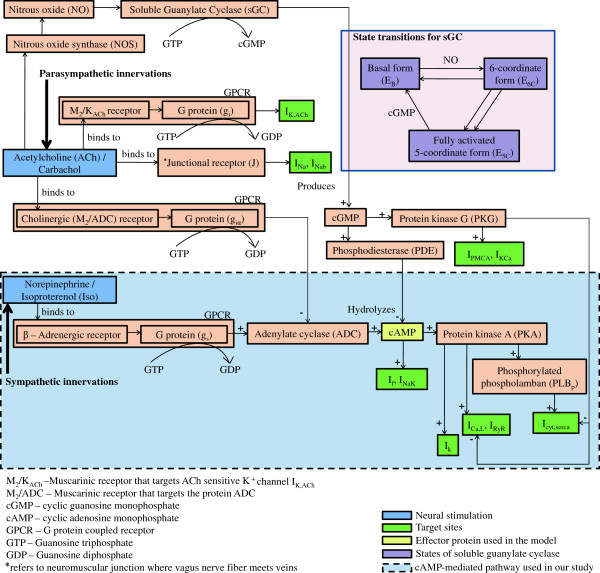
**cAMP and cGMP pathways.** Reaction pathways triggered by sympathetic and parasympathetic neural stimuli invoking cAMP [25] and cGMP-mediated [26] modulation of various sub-cellular targets respectively. ACh-mediated effects of junctional receptor on *Na*^+^ current and background *Na*^+^ current and extrajunctional muscarinic M _2_/K _*ACh*_ receptor on I _*K*,*ACh*_ (direct muscarinic pathway) and cAMP-mediated effects of *β*-adrenoreceptor (adrenergic pathway) and cholinergic M _2_/ADC receptor (indirect muscarinic pathway) on L-type *Ca*^2+^ (I _*Ca*,*L*_), *K*^+^ (I _*K*_), hyperpolarization-activated (I _*f*_), and *Na*^+^/ *K*^+^ (I _*NaK*_)currents are shown *‡*. Although cAMP-dependent modulation of I _*Ca*,*L*_ and *I*_*up*_ are known to be PKA-mediated, we make use of a lumped cAMP term to model this influence. A similar lumped cAMP term is employed to modulate phosphorylation of PLB which in turn affects the SERCA pump. ACh triggered NO-mediated synthesis of cGMP by sGC is modeled employing a 3-state model for sGC transition based on Yang et al. [26]. cGMP is involved in suppressing cAMP activity via PDE. cGMP also enhances *I*_*PMCA*_, *Ca*^2+^ activated *K*^+^ channel (*I*_*KCa*_) and suppresses *I*_*Ca*,*L*_, *I*_*cyt*,*serca*_ via PKG (not explicitly modeled, but lumped into a cGMP term). *‡*The rat ventricular cell model used in this study is however limited to *Ca*^2+^ related channel, exchanger and pumps (*I*_*Ca*,*L*_, *I*_*NaCa*_, *I*_*PMCA*_ and *I*_*cyt*,*serca*_), while lacking exclusive *Na*^+^ or *K*^+^ related channels and transporters (as shown in Figure 2, Krishna et al. [15]). The part of the model describing cAMP-mediated pathway used in our study is highlighted (blue).

We incorporate the effects of *β*-adrenergic stimulation via cAMP-dependent modulation based on a model (Figure 2) derived from Demir et al. [25]. Stimulation of *β*-adrenoceptors by Isoproterenol (Iso) results in the activation of a G protein (g _*s*_) that stimulates Adenylate cyclase (ADC) and enhances the production of cAMP. Subsequently, cAMP may directly or indirectly activate various intracellular targets including ion channels and exchangers. The indirect modulation involves activation of cAMP-dependent Protein kinase A (PKA) before modulation of the channel protein. The reaction kinetics for the cGMP-mediated pathway (Figure 2) involving acetylcholine (ACh), nitric oxide (NO) and soluble guanylate cyclase (sGC) are adopted from Yang et al. [26]. Although it is well known that cGMP modulates its targets via protein kinase G (PKG) or phosphodiesterase (PDE), we have refrained from modeling these protein interactions. Given that NO synthase inhibition and/or NO donor had little or only marginal effects on the FFR in rat ventricular myocardium [27], the cGMP level is kept constant in this study.

### DHP-sensitive C*a*^2+^ channel

Upon cell depolarization, the L-Type *Ca*^2+^ channel (*I*_*Ca*,*L*_) brings trigger *Ca*^2+^ into the dyad that facilitates *Ca*^2+^ release from the apposed Ry-sensitive *Ca*^2+^ channel associated with the membrane of the junctional sarcoplasmic reticulum (jSR). Feedback controlled interaction between these apposed channels is critical in CICR as well as in the maintenance of the overall cellular *Ca*^2+^ balance. Besides membrane voltage, gating of the *I*_*Ca*,*L*_ channel is also influenced by two prominent *Ca*^2+^-mediated effects, namely *Ca*^2+^-dependent inactivation (CDI) and *Ca*^2+^-dependent facilitation (CDF).

**Figure 3:**
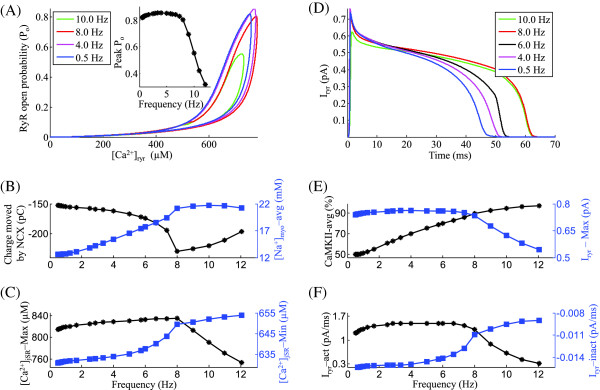
***I***_***ryr***_**.** Frequency-dependent characteristics of the SR *Ca*^2+^ release channel *I*_*ryr*_. Voltage clamp protocol used is a 50 ms step pulse to 10 mV from a holding potential of -40 mV. **(A)** RyR open probability vs [ *Ca*^2+^] _*ryr*_ for increasing frequency of stimulation (0.5 Hz to 10.0 Hz). The inset shows the frequency-dependent modulation of peak RyR open probability. **(B)** Frequency dependence of average charge transported by NCX and average [ *Na*^+^] _*myo*_ in the myoplasm. **(C)** Rate dependence of pre-release maximum and post-release minimum SR *Ca*^2+^ level. **(D)** Traces for RyR release for the corresponding frequencies of stimulation in panel A. **(E)** Dependence of average level of activated-CaMKII and peak *I*_*ryr*_ current on frequency of stimulation. **(F)** Frequency-dependent changes in average *I*_*ryr*_ activation and inactivation rates. The legend for traces in panel A is shared by panel D.

In CDI, *Ca*^2+^ that enters the dyad either via the *I*_*Ca*,*L*_ channel or through RyR release from the jSR binds to the protein Calmodulin (CaM) which is tethered to the C-terminus of the *I*_*Ca*,*L*_ channel [28], modulating the interaction of CaM with the *Ca*^2+^ channel. Leucine-Alanine (LA) and Isoleucine-Glutamine (IQ) are 2 adjacent motifs in the *Ca*^2+^ sensing domain of the C-terminus of the *I*_*Ca*,*L*_ channel. A *Ca*^2+^-dependent switch of CaM from LA to IQ motif removes CaM from the inner mouth of the channel pore, thus causing an enhancement in inactivation by facilitating the constriction of the pore [29,30]. CDI is a critical negative feedback mechanism which causes decreased *Ca*^2+^ entry via *I*_*Ca*,*L*_ when the SR load is high with an accompanying large myoplasmic *Ca*^2+^ transient, and it results in increased *Ca*^2+^ entry via *I*_*Ca*,*L*_ when [ *Ca*^2+^] _*myo*_ is small due to a low SR load. We utilize a 2-state model, as shown in Figure 3A of our previous study Krishna et al. [15], to simulate CDI.

In contrast, *Ca*^2+^-dependent facilitation (CDF) is a *Ca*^2+^/CaM-mediated enhancement in *I*_*Ca*,*L*_ via activation of CaMKII [31], which has been shown to tether to the *I*_*Ca*,*L*_ channel [32], functioning as a *Ca*^2+^ signalling sensor for facilitation. Activated Calcineurin (CaN) is also observed to facilitate *Ca*^2+^ entry via the *I*_*Ca*,*L*_ channel [33]. CDF is implicated in causing a gradual increase in amplitude and an accompanying decrease in inactivation over consecutive pulses after a resting interval [34]. Enhancement of *I*_*Ca*,*L*_ caused by activated-CaMKII and CaN plays a key role in negating the effects of incomplete *I*_*Ca*,*L*_ channel recovery at faster heart rates, thus helping to improve cardiac performance during exercise. Although CDF and CDI of *I*_*Ca*,*L*_ coexist, CDI responds much faster (within the same beat) than CDF (over several beats). Our model incorporates CDF by allowing the rate constants in the 6-state Markovian model of the *I*_*Ca*,*L*_ channel ($k_{24}^{\text{dhpr}}$, $k_{25}^{\text{dhpr}}$ and $k_{12}^{\text{dhpr}}$ in Figure 3A, Krishna et al. [15]) to be a function of the available active CaMKII and CaN.

Rate-dependent increases in *I*_*Ca*,*L*_ can also be caused by frequency-dependent increase in *β*-adrenergic stimulation increasing the level of available cAMP (Table 1), which in turn causes an enhancement in *I*_*Ca*,*L*_ channel current via protein kinase-A (PKA). Although PKA is involved in the indirect regulation of the *I*_*Ca*,*L*_ channel, its effect is considered lumped into the conductance term in the ionic current description (Appendix A3, Equations 1-2). The effect of *β*-adrenergic stimulation via cAMP, particularly its dose-dependent influence on L-Type *Ca*^2+^ channels both in terms of modifying the single channel behavior such as *Ca*^2+^ ion permeability as well as overall channel recruitment characteristics, is not clearly understood. While cAMP has been shown to increase channel open probability [35,36], increased levels of cAMP also result in increased phosphorylation of L-type *Ca*^2+^ channels, causing an increased permeability to *Ca*^2+^ ions [36-38].

**Table 1:** Frequency dependence of intracellular cAMP concentration

**Stimulation frequency (Hz)**	**Intracellular cAMP concentration (*μM*)**
0.50 - 1.67	2.67 ^*δ*^
2.50	3.00
2.86	3.19
3.33	3.45
4.00	3.85
5.00	4.67
5.71	5.58
6.67	18.06
8.00	24.58
10.00	27.03
12.00	29.17

*δ*- Value used under basal conditions. Intracellular cAMP concentration corresponding to maximal *β*-adrenergic stimulation at various stimulation frequencies used in the model (based on Equation 2, Demir et al. [25]).

### Ry-sensitive C*a*^2+^ release channel

Ry-sensitive *Ca*^2+^ channels on the jSR membrane respond to the trigger *Ca*^2+^ entering the dyadic space via the *I*_*Ca*,*L*_ channel on the plasma membrane. A larger *Ca*^2+^ release from the jSR follows, forming the basis for *Ca*^2+^ induced *Ca*^2+^ release (CICR) and subsequent contraction of the ventricular cell. CaM that is tethered to the Ry-sensitive *Ca*^2+^ channel [39] facilitates frequency-dependent CaMKII and CaN-assisted modulation of that channel. Although CaMKII is known to bind to the RyR [40-42], the effect of this association has not yet been resolved. In lipid bilayer studies, CaMKII has been shown to increase [40,41,43] or decrease [44] RyR open probability. In studies on rat ventricular myocytes, it has been shown that endogenous CaMKII has an activating effect on the RyR *Ca*^2+^ release channel [45-48]. However, a contrasting study shows that constitutively active CaMKII depresses RyR release [49]. Thus, the functional consequence of phosphorylation of RyR by CaMKII remains controversial. Since the bulk of the published literature on this topic points toward an activating effect of CaMKII on RyR, this concept is adopted in our model by making the rate constants in our 4-state Markovian model of the RyR channel ($k_{12}^{\text{ryr}}$, $k_{41}^{\text{ryr}}$, $k_{43}^{\text{ryr}}$ and $k_{32}^{\text{ryr}}$ in Figure 3B, Krishna et al. [15]) functions of the available active CaMKII. Although CaN is reported to regulate ryanodine receptor *Ca*^2+^ release channels in rat heart [50], we have refrained from modeling its influence on the RyR channel because CaN is known to be constitutively active in the dyad [2] exhibiting only minor frequency-dependent modulation in its level, hence making its rate-dependent regulatory role insignificant.

### SERCA pump

In rat ventricular myocytes, 85-90% [51,52] of the systolic increase in *Ca*^2+^ in the myoplasm is recovered back into the SR stores via the sarcoplasmic reticulum *Ca*^2+^-ATPase (SERCA) pump. Frequency-dependent CaMKII activity is known to cause an acceleration of relaxation [3,23]. CaMKII affects the SERCA pump via direct phosphorylation, assisting in enhancement of SR *Ca*^2+^ transport by increasing the pumping rate [4]. This feature is incorporated in our model by letting the rate constants for *Ca*^2+^ binding to/release from the SERCA pump depend on the available active CaMKII. The SERCA pump is also indirectly affected by CaMKII via phosphorylation of unphosphorylated phospholamban (PLB), relieving the inhibition caused by PLB on the SERCA pump and thereby increasing the sensitivity of the pump for *Ca*^2+^ uptake. This indirect effect is modeled by having the rate constant for phosphorylation of PLB be a function of active CaMKII in the myoplasm. These two effects cause enhancement in SR *Ca*^2+^ uptake in an activity-dependent fashion. Expression of CaN, a protein that is a phosphatase, paradoxically has been reported to cause an increased level of PLB phosphorylation via an unknown indirect mechanism in transgenic mice. In CaN knock-out mice, decreasing phosphorylation of PLB allowed an increased level of inhibition of the SERCA pump, which resulted in poor muscle contraction and relaxation [5,6,53]. However, it is unclear if this behavior is a result of other compensatory mechanisms such as decreased CaMKII expression or enhanced PLB to SERCA ratio. On the contrary, CaN has been reported to inhibit SERCA activity in isolated non-failing human myocardium [54] in-vitro. Hence, we model the role of CaN in rate-dependent inhibition of the SERCA pump via PLB dephosphorylation by allowing the rate constant for phosphorylation of PLB to be dependent on available active CaN in the myoplasm. With increasing heart rates, *β*-adrenergic stimulation results in increasing levels of cAMP in vivo (Table 1 shows values for maximal *β*-adrenergic stimulation), which in turn phosphorylates PLB via PKA, causing enhanced uptake by the SERCA pump (Appendix A3, Equations 5-6). Together, activity-dependent recruitment of these CaMKII and cAMP-mediated effects at high frequencies counter the effect of CaN as well as decreasing cardiac cycle duration on SR refilling.

### Electro-mechanics

Our model for cardiac contractile mechanics is based on the approximate model of cooperative activation and crossbridge cycling reported by Rice et al. [55] with the following modifications: (a) the first-order rate constants for the transformation of the troponin/tropomyosin regulatory complex (outside the single overlap region between the thick and thin filaments) from a crossbridge non-permitting state to a crossbridge permitting state and vice-versa are chosen as 500 s-1 and 50 s-1 respectively in order to reproduce results reported by Rice et al. [55]; (b) the *β*-adrenergic agonist isoproterenol (ISO) is known to cause a decrease in myofilament *Ca*^2+^ sensitivity as a result of PKA mediated phosphorylation of troponin I [51,56] at Ser23/Ser24. Specifically, a two-state Markovian model is added to allow cAMP-dependent PKA-mediated interaction between troponin I (TnI) and the *Ca*^2+^-binding regulatory site on troponin. As reported by Messer et al. [57], the unphosphorylated form of TnI (TnI _*u*_) modulates the *Ca*^2+^ affinity of the regulatory site on troponin. We model the effects of cAMP by allowing the cumulative activation rate constant for *Ca*^2+^-binding to the troponin regulatory site to be a function of unphosphorylated TnI _*u*_, the availability of which is in turn dependent on the amount of [cAMP] present (Appendix, Equations 10,12); (c) the large Q_10_ values used by Rice et al. (Qf _*app*_, Qh _*f*_, Qh _*b*_ and Qg _*xb*_, Table 1, [55]) are decreased from 6.25 to 2.25 in order to reproduce temperature dependence of peak force developed in intact thin rat ventricular trabeculae [58]. The rate constants (Appendix, Equations 13,17) governing cross-bridge kinetics are modeled as functions of [cAMP] to reproduce stimulation frequency dependent increase in contraction and relaxation rates. Although a calmodulin (CaM) mediated pathway has been reported [59] to be responsible for modulation of myofibrillar *Ca*^2+^-sensitivity (implying a possible CaM mediated role for Ca-dependent kinases or phosphatases in regulating myofilament contractility, particularly in frequency dependent acceleration of relaxation), we refrain from modeling this effect as the molecular mechanisms involved remain unresolved.

## Results

From our modeling standpoint, the dyadic coupling unit (DCU) as defined by Krishna et al. [15] is a fundamental element involved in the mechanism of CICR. These previous studies have described the control features of this unit, as well as its interaction with the SERCA pump and free sarcolemmal pumps and exchangers to achieve a homeostatic regulation of myoplasmic *Ca*^2+^ concentration. We now extend our voltage clamp studies to address the subject of frequency-dependent characteristics of CICR and begin with the study of frequency dependence of the DCU, one of the most important components of the model. All the frequency-dependent behavior discussed here is at steady state (150 cycles) unless otherwise specified. The first task is to examine the frequency dependence of the *I*_*Ca*,*L*_ trigger current, followed by the RyR *Ca*^2+^ channel, highlighting the rate-dependent CaM-mediated signalling involved. This analysis is followed by examining the SR *Ca*^2+^ content and the various factors controlling it in a rate-dependent manner. A quantitative study of the overall cellular *Ca*^2+^ balance is performed to highlight its rate-dependent feature. Emphasis is placed on relative roles played by the longitudinal sarcoplasmic reticulum (LSR) membrane SERCA pump and the plasma membrane *Na*^+^/ *Ca*^2+^ exchanger, as two principal *Ca*^2+^ transport routes in the maintenance of *Ca*^2+^ homeostasis. We finally examine the myoplasmic *Ca*^2+^ transient as a function of frequency with a particular interest in the rate dependence of the force-frequency response (FFR) generated by the coupled electromechanical model. This is subsequently followed by an investigation of the rate-dependent influence of cAMP-mediated *β*-adrenergic stimulation on the cardiac contractile response.

### L-type C*a*^2+^ current (*I*_*Ca*,*L*_)

**Figure 4:**
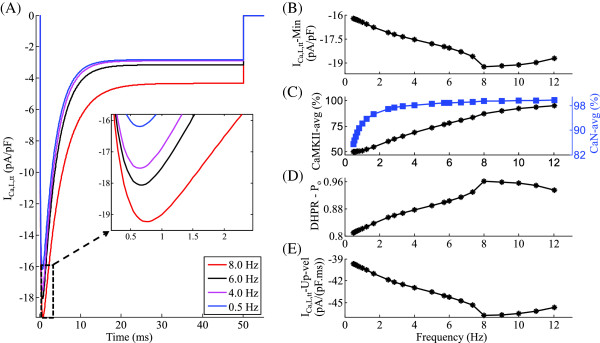
***I***_***Ca*,*L***_**.** Frequency-dependent characteristics of the DHP-sensitive *Ca*^2+^ channel *I*_*Ca*,*L*_, which forms the trigger current. Voltage clamp protocol used is a 50 ms step pulse to 10 mV from a holding potential of -40 mV. **(A)***I*_*Ca*,*L*_ at different frequencies of stimulation ranging from 0.5 Hz to 8.0 Hz. Inset gives an expanded view of the rate-dependent modulation of *I*_*Ca*,*L*_ over the range of frequencies investigated **(B)** Dependence of peak *I*_*Ca*,*L*_ current on frequency of stimulation **(C)** Frequency-dependent increase in average levels of activated auxiliary proteins CaMKII and CaN in the dyad **(D)** Frequency-dependent modulation of peak open probability of the DHP-sensitive *Ca*^2+^ channel. **(E)** Rate-dependent changes in *I*_*Ca*,*L*_ upstroke velocity.

An increase in stimulation frequency from 0.5 Hz to 8 Hz, results in a frequency-dependent monotonic increase in the peak trigger current (inset in Figure 4A and Figure 4B) while slowing down the rate of decline after it reaches its maximum (note the traces corresponding to 0.5 Hz to 8 Hz in Figure 4A). The most critical mechanism involved in frequency encoding of the *I*_*Ca*,*L*_ channel activity is the rate-dependent change in the average level of activated-CaMKII (Figure 4C), which is known to assist CaM-mediated *Ca*^2+^-dependent facilitation (CDF). As stimulation frequency is increased from 0.5 Hz to 8 Hz, the increase in peak *I*_*Ca*,*L*_(Figure 4B) closely tracks the increase in the average level of activated-CaMKII. However, beyond 8 Hz a decrease in peak is observed (Figure 4B) despite a further increase in CaMKII (Figure 4C). This occurs as a result of incomplete channel recovery at high (> 8Hz) stimulation rates, which results in a decline in peak channel open probability (Figure 4D). At low (0.5 Hz ≤ f ≤ 4.0 Hz) stimulation rates, an increase in activated CaN (85% to 97%) is also known to enhance *I*_*Ca*,*L*_ channel activity, whereas at higher (> 4 Hz) rates, the lack of a substantial rate-dependent increase in its average level (Figure 4C) minimizes its role in *Ca*^2+^-dependent facilitation. The maximum value attained by the open probability of the DHP-sensitive *Ca*^2+^ channel (Figure 4D) reflects the trend shown by the peak value of the trigger current over the entire range of stimulation frequencies (0.5 to 12.0 Hz) investigated. At frequencies less than 8 Hz, the upstroke velocity of *I*_*Ca*,*L*_ current can be seen to increase with an increase in frequency, but above 8 Hz it begins to decline (Figure 4E) due to insufficient time for full channel recovery. It is important to note that the model predicts a frequency-dependent modulation in peak *I*_*Ca*,*L*_ current of less than 20% over the entire frequency range (0.5 Hz to 12.0 Hz). This small modulation of peak current (Figure 4B) is far less than the percentile changes in CaMKII activation (50%, Figure 4C), due to the insufficient time for channel recovery at high (> 4 Hz) stimulation rates.

### RyR C*a*^2+^ release

Figure 3A shows a phase plot of RyR open probability (*P*_*o*_) versus [ *Ca*^2+^] _*RyR*_ constructed from model-generated data corresponding to different stimulation rates with the inset showing the peak open probability attained by each of the phase loops. As the stimulation frequency is increased from 0.5 Hz to 4 Hz, a marginal increase (< 1%) in peak RyR open probability occurs (inset in Figure 3A) as a result of the factors: (a) a frequency-dependent CaMKII mediated (Figure 3B) *I*_*Ca*,*L*_ facilitation (Figure 4A); (b) a moderate increase in SR *Ca*^2+^ content (despite a relatively constant maximal SERCA uptake rate), due to increased trigger current and the subsequent increase in [ *Ca*^2+^] _*myo*_ combined with an increase in [ *Na*^+^] _*myo*_ (30% in Figure 3B) which impedes *Ca*^2+^ extrusion via the NCX; and (c) a direct CaMKII-mediated enhancement in RyR release. As the stimulation frequency is increased from 4 Hz to 8 Hz, despite a moderate increase in both the trigger current and SR *Ca*^2+^ content, a small decrease in RyR open probability occurs as a result of an increase in post-release [ *Ca*^2+^] _*jSR*_ (Figure 3C), which forces an incomplete luminal sensor-based RyR recovery (as described in Krishna et al. [15]). Beyond 8.0 Hz, the peak RyR open probability decreases due to two mechanisms: (a) a small parallel decrease in the trigger current (Figure 4B) indicating a close coupling enforced by a stable CICR, and (b) insufficient time for full channel recovery, accompanied by a falling pre-release diastolic jSR *Ca*^2+^ level (Figure 3C). Both the declining trigger current and the declining [ *Ca*^2+^] _*jSR*_ result in a strong decline in [ *Ca*^2+^] _*RyR*_. The decrease in [ *Ca*^2+^] _*RyR*_ at high (> 8 Hz) frequencies can be seen in Figure 3A, where the open probability loop for 10 Hz is enclosed within that of 8 Hz. The frequency dependence of the area enclosed by the loops (which indicates the amount of SR *Ca*^2+^ released into the dyad) mirrors that of the peak RyR open probability. The SR *Ca*^2+^ release from the Ry-sensitive receptor exhibits strong frequency-dependent behavior as shown in Figure 3D. Instantaneous RyR flux is obtained by multiplying the open probability by the concentration gradient across the channel. The rate dependence of the peak instantaneous RyR flux (Figure 3D, E) is a result of two factors: (a) the frequency dependence of peak RyR open probability (Fig 3A), which indicates the degree of recruitment of RyR release channels; and (b) the pre-release SR *Ca*^2+^ content (Figure 3C), which establishes the initial concentration gradient across the release channel. Figure 3D also shows the gradation in the time required for RyR recovery.

The frequency dependence of RyR release activation (Figure 3F), shares its characteristics with the peak RyR open probability (Figure 3A). Besides increasing trigger current, RyR release is also facilitated by increasing levels of activated-CaMKII (Figure 3E) and increasing SR *Ca*^2+^ content(Figure 3C). The frequency-dependent modulation of RyR inactivation rate (Figure 3F) mimics the rate dependence of minimum SR *Ca*^2+^ levels reached after release, mediated via the luminal sensor. With increasing stimulation frequency (0.5 Hz ≤ f ≤ 8.0 Hz), despite decreasing time available for uptake, increasing pre-release SR *Ca*^2+^ content is achieved only by increased rate of SR filling. This increased rate of SR filling translates into a faster decline in inhibition via the luminal sensor (a RyR inactivation mechanism described in Krishna et al. [15]). Hence, the rate of inactivation decreases as the frequency is increased from 0.5 Hz to 8.0 Hz (Figure 3F) owing to increasing SR *Ca*^2+^ content in this range of frequencies. With increase in frequency, the rate-dependent increase in the level of activated-CaMKII in the dyadic space also causes an increased CaMKII-mediated upregulation of the ryanodine receptor [40,41,43,45-48]. This CaMKII mediated up-regulation delays the onset of declining SR *Ca*^2+^ content-driven (8.0 Hz ≤ f ≤ 12.0 Hz) increase in the rate of RyR channel inactivation. The RyR channel experiences a frequency-dependent modulation by both the trigger current and the amount of activated-CaMKII on its dyadic side. On its luminal side, it experiences modulation by the luminal sensor (as described in Krishna et al. [15]) controlling refractoriness, and the SR *Ca*^2+^ content providing the drive for *Ca*^2+^ through the channel.

### SR C*a*^2+^ content

**Figure 5:**
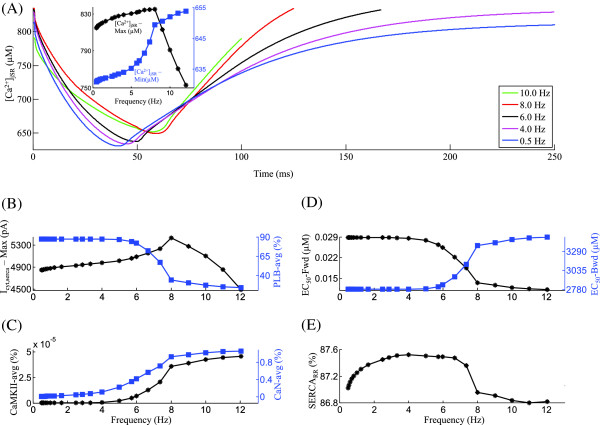
**[*Ca***^**2+**^**] **_***jSR***_**.**Mechanisms underlying the frequency-dependent modulation in SR *Ca*^2+^ content. Voltage clamp protocol used is a 50 ms step pulse to 10 mV from a holding potential of -40 mV. **(A)** [ *Ca*^2+^] _*jSR*_ traces indicating rate-dependent changes in SR filling. The inset shows the frequency dependence of the maximum and minimum SR *Ca*^2+^ concentration. **(B)** Frequency dependence of the maximal pumping rate of SERCA and the average level of phosphorylated PLB. **(C)** Frequency-dependent increase in average levels of activated auxiliary proteins CaMKII and CaN in the myoplasm. **(D)** Frequency dependence of the half-activation constant for the forward and backward flow via the SERCA pump. **(E)** Rate dependence of the relative role of the SERCA pump (when compared to the *Na*^+^/ *Ca*^2+^ exchanger (*I*_*NaCa*_)and the plasma membrane *Ca*^2+^ ATPase pump (*I*_*PMCA*_).

The frequency dependence of pre-release *Ca*^2+^ level in the SR is a result of several factors namely: (a) available [ *Ca*^2+^] _*myo*_ for sequestration; (b) the rate-dependent behavior of the SERCA pump; (c) the frequency dependence of the release characteristics of Ry-sensitive *Ca*^2+^ channel; and (d) the cumulative transmembrane *Ca*^2+^ transport via *I*_*Ca*,*L*_ and NCX. The combined effect of these factors results in the biphasic relationship (inset in Figure 5A) between free *Ca*^2+^ content in the SR and the frequency of stimulation. As the frequency is increased from 0.5 Hz to 8.0 Hz, the SR *Ca*^2+^ content increases due to the following frequency-dependent, active CaMKII-mediated effects: (a) enhancement of maximal uptake rate of the SERCA pump due to increase in PLB phosphorylation (Figure 5B) assisted by increasing levels of activated-CaMKII (Figure 5C); (b) decrease in the half-activation constant for the forward (myoplasm to SR) operation of the SERCA pump (Figure 5D) translating into increased *Ca*^2+^ sensitivity of the pump for uptake; and (c) increase in half-activation constant for the backward (SR to myoplasm) operation of the SERCA pump (Figure 5D), reducing tendency for back-flow via the SERCA pump. The increase in SR *Ca*^2+^ content is also facilitated by a CaMKII mediated enhancement in *I*_*Ca*,*L*_ as well as inhibition of *Ca*^2+^ extrusion via NCX due to increasing [ *Na*^+^] _*myo*_. As the stimulation frequency is gradually increased further from 8.0 Hz to 12.0 Hz, a steep decrease in SR *Ca*^2+^ content is observed (inset in Figure 5A). This frequency-dependent decrease in pre-release SR *Ca*^2+^ content at very high (> 8.0 Hz) stimulation frequencies is a result of the following: (a) a decrease in maximal uptake rate of the SERCA pump (Figure 5B) along with a significant decrease in time available for resequestration of cytosolic *Ca*^2+^; (b) a decrease in *Ca*^2+^ entry into the cell via the trigger current *I*_*Ca*,*L*_ (Figure 4B); and (c) relatively constant intracellular [ *Na*^+^] _*myo*_ (Figure 3B). Between stimulation frequencies 0.5 Hz and 8.0 Hz, the characteristics of the post-release SR *Ca*^2+^ level tracks the pre-release peak SR *Ca*^2+^ content. However, beyond 8.0 Hz, a declining SR *Ca*^2+^ content causes increasing inhibition on RyR release via the luminal sensor [15], resulting in a gradual increase in post-release SR *Ca*^2+^ level.

The frequency encoding involved in the uptake mechanism via the SERCA pump is strongly regulated by two key proteins, PLB and activated-CaMKII. The rate-dependent decrease in the level of unphosphorylated phospholamban (PLB) (Figure 5B) capable of inhibiting the SERCA pump is a result of increasing levels of activated-CaMKII (Figure 5C). It is important to note that phosphorylation of PLB is sensitive to small changes in activated-CaMKII [23]. Our results show that activated-CaMKII (Figure 5C) has a prominent role in rate-dependent modulation of the SERCA pump despite its low level (three orders of magnitude compared to activated CaN (note the 10 ^−5^ on left ordinate in Figure 5C)) in the myoplasm [2]. Our results show that activated CaN (Figure 5C) has minimal role in rate-dependent inhibition of the SERCA pump. However, over-expression of CaN in failing heart is known to significantly compromise SERCA activity [54]. At a given frequency of stimulation, an increase in trigger current forces a gradual increase in SR *Ca*^2+^ uptake which in turn results in a corresponding increase in RyR release, which translates into an elevated cytosolic *Ca*^2+^ level and subsequently an increased activated-CaMKII level. This causes an increase in SR *Ca*^2+^ uptake rate via phosphorylation of PLB, thus completing a positive feedback loop. Hence, the peak *I*_*Ca*,*L*_ trigger current sets the peak SR *Ca*^2+^ content if the SERCA pump is not operating in saturation. Thus the frequency dependence of the maximal rate of uptake by the SERCA pump (Figure 5B) shares its characteristics with peak *I*_*Ca*,*L*_ current (Figure 4B) linked together by activated-CaMKII (Figure 5C). It is important to note that rate-dependent enhancement in SERCA activty by CaMKII significantly overrides the inhibitory influence of CaN in a non-failing heart [54]. Figure 5B also shows the underlying rate-dependent decline in unphosphorylated PLB responsible for rate-dependent increase in SR uptake.

Although marginally (< 1% change over 0.5 Hz ≤ f ≤ 12.0 Hz) frequency-dependent, the SERCA pump has a primary role in resequestration of myoplasmic *Ca*^2+^ when compared with the combined influence of *Na*^+^/ *Ca*^2+^ exchange (*I*_*NaCa*_) as well as *Ca*^2+^ transport via the plasma membrane *Ca*^2+^ ATPase pump,(*I*_*PMCA*_). As the frequency of stimulation is increased from 0.5 Hz to 4.0 Hz, increasing activated-CaMKII-dependent enhancement in SERCA activity assisted by a decrease in *Ca*^2+^ extrusion via NCX due to increasing [ *Na*^+^] _*myo*_ (Figure 3B) result in a marginal increase in the relative role of SERCA pump as shown in Figure 5E. However, as frequency is further increased, declining SERCA-mediated uptake activity due to a decrease in CaMKII-mediated SERCA enhancement, as well as reduced time available for uptake results in a small negative slope to the frequency dependence of the relative role of SERCA pump (Figure 5E).

### Myoplasmic Ca^2+^ transient [Ca^2+^]_myo_

**Figure 6:**
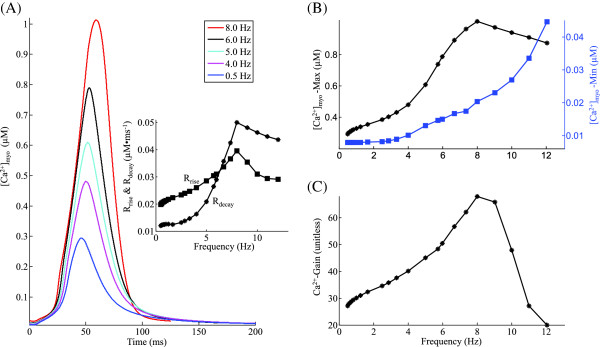
**[*Ca***^**2+**^**] **_***myo***_**.** Frequency-dependent modulation of myoplasmic *Ca*^2+^ transient. Voltage clamp protocol used is a 50 ms step pulse to 10 mV from a holding potential of -40 mV. **(A)** [ *Ca*^2+^] _*myo*_ traces showing rate-dependent effects on systolic *Ca*^2+^ concentration in the myoplasm for increasing stimulation frequencies from 0.5 Hz to 8.0 Hz. The inset shows the change in the maximum rate of rise and decay of the [ *Ca*^2+^] _*myo*_ transient with stimulation frequency. **(B)** Frequency dependence of the maximum and minimum myoplasmic *Ca*^2+^ concentration. **(C)** Bi-phasic rate dependence of EC coupling gain.

Figure 6A shows the traces of [ *Ca*^2+^] _*myo*_ at increasing frequencies showing an increase in the peak myoplasmic *Ca*^2+^ transient during systole with increasing stimulation frequency in the range 0.5 Hz to 8.0 Hz. A ’primary phase’ negative peak FFR (a decrease in peak contractile force with increase in stimulation frequency observed at low (< 1.0 Hz) stimulation rates) reported in studies on rat ventricular myocytes [8,9,60,61] could be a result of low unphysiological temperatures employed in those studies. The inset in Figure 6A shows that the peak rate of rise (R _*rise*_) and decay (R _*decay*_) of the [ *Ca*^2+^] _*myo*_ transient increases with the frequency of stimulation from 0.5 Hz to 8.0 Hz. The increase in rate of rise is a result of an increase in peak trigger current *I*_*Ca*,*L*_ (Figure 4B) and SR *Ca*^2+^ content (inset in Figure 5A) while the increase in rate of decay is a result of CaMKII-mediated acceleration of relaxation (Figure 5C) due to a rate-dependent enhancement in uptake by the SERCA pump. An opposite effect is observed in the rate of rise and decay of the [ *Ca*^2+^] _*myo*_ transient beyond a stimulation rate of 8.0 Hz due to declining peak trigger current and reduced CaMKII activity. As shown in Figure 6B, increasing stimulation rate from 0.5 Hz to 8.0 Hz results in a steep increase in peak [ *Ca*^2+^] _*myo*_ (maximum), which parallels a strong increase in pre-release diastolic [ *Ca*^2+^] _*jSR*_ (Figure 5A). This increase in [ *Ca*^2+^] _*jSR*_ is mirrored by a corresponding increase in pre-release minimum value attained by [ *Ca*^2+^] _*myo*_ during diastole (Figure 6B). As the stimulation frequency is increased beyond 8.0 Hz, although the SERCA activity is saturated [12], the declining SR *Ca*^2+^ content (inset in Figure 5A) aids in the decrease of peak [ *Ca*^2+^] _*myo*_ by compromising SR release. However, a lack of sufficient time for uptake continues to manifest in increasing pre-release diastolic [ *Ca*^2+^] _*myo*_ (Figure 6B) at these rates. Our study suggests that a decrease in pH _*i*_ causing a decrease in myofilament *Ca*^2+^ sensitivity [62] is not required to cause the ’secondary phase’ negative peak FFR (a decrease in peak contractile force with increase in stimulation frequency observed at high (> 8.0 Hz) stimulation rates). We attribute this phase which involves a decrease in force response, to the declining peak [ *Ca*^2+^] _*myo*_ as seen in Figure 6B. The rate-dependence of peak [ *Ca*^2+^] _*myo*_ for increasing stimulation frequencies (0.5 Hz ≤ f ≤ 12.0 Hz) is comparable to that reported by Kentish et al. (Figure 1, [63]) in rat ventricular trabeculae. Figure 6C shows the frequency-dependent changes in gain calculated as the ratio of peak *Ca*^2+^ transient in the presence of CICR to the peak calcium transient in its absence, contributed by the trigger calcium alone [64]. The EC coupling gain increases with the frequency of stimulation from 0.5 Hz to 8.0 Hz, due to rate dependent CaMKII-mediated increase in uptake rate of the SERCA pump (Figure 5B), which decreases the peak [ *Ca*^2+^] _*myo*_ in the absence of CICR and increases peak [ *Ca*^2+^] _*myo*_ in its presence (due to increase in SR content (inset in Figure 5A)). Beyond a stimulation frequency of 8.0 Hz, the rapid decrease in SR content causes a steep decline in EC coupling gain.

### Positive force-frequency relationship

**Figure 7:**
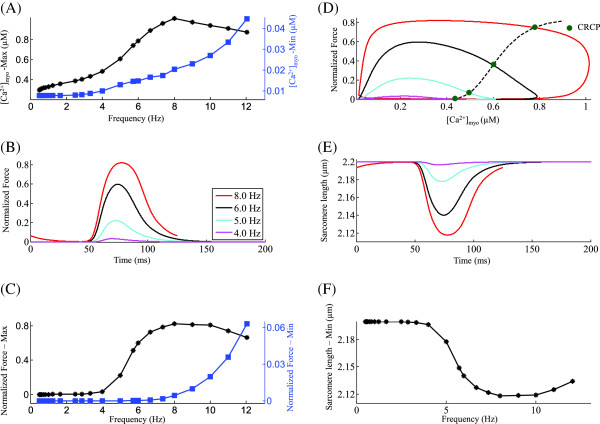
**Contractile force and sarcomere length.** Rate-dependent changes in myoplasmic *Ca*^2+^ concentration, the corresponding contractile force generated and the resulting sarcomere length. Voltage clamp protocol used is a 50 ms step pulse to 10 mV from a holding potential of -40 mV. **(A)** Frequency dependence of the maximum and minimum myoplasmic *Ca*^2+^ concentration **(B)** Traces for normalized isometric force developed at stimulations rates of 4, 5, 6, and 8 Hz. **(C)** Rate dependence of the maximum and minimum normalized force generated. **(D)** Phase loops for normalized isometric force vs [ *Ca*^2+^] _*myo*_ for increasing frequency of stimulation (4.0 Hz to 8.0 Hz) corresponding to panel B. **(E)** Traces for sarcomere contraction corresponding to isometric force in panel B. **(F)** Frequency dependence of minimum sarcomere length achieved at peak contraction. The legend for traces in panel B is shared by panel D and E.

Changing the stimulation rate has a very prominent effect on cardiac muscle contraction by way of affecting the amplitude and time course of the intracellular *Ca*^2+^ transient (Figures 6 and 7A). Here, we simulate a positive peak force-frequency response elicited using a voltage clamp stimulation protocol. Figure 7B shows the traces for the isometric contractile force generated for increasing stimulation frequencies. As stimulation frequency increases from 0.5 Hz to 8.0 Hz, peak contractile force increases (Figures 7B and C) mirroring an increase in peak [ *Ca*^2+^] _*myo*_. As seen in Figures 4B and 5B, this is mainly a result of CaMKII-dependent upregulation of *I*_*Ca*,*L*_ and SERCA activity which translates into a frequency-dependent increase in SR *Ca*^2+^ content, and thereby SR *Ca*^2+^ release. As reported in the literature [55], the non-linear sigmoidal steady state Force- *Ca*^2+^ relationship (overlayed in Figure 7D) causes a non-linear response in peak force developed for a corresponding change in peak [ *Ca*^2+^] _*myo*_. As shown in Figure 7D the contraction-relaxation coupling point (CRCP) moves to larger values of force and *Ca*^2+^ concentration with increase in rate of stimulation. The peak contractile force is attained at a physiological rate of 8.0 Hz, beyond which the gradual decline in peak *I*_*Ca*,*L*_ current (Figure 4B), which forces a corresponding decrease in SR *Ca*^2+^ content (Figure 5A), and hence RyR release (Figure 3E), causes a marginal decline in peak contractile force generated. The frequency at which peak contractile force is generated is temperature-dependent as reported by Layland et al. [12] and shifts to the right on the frequency axis as the temperature increases. The peak force frequency response obtained mimics the experimentally observed frequency dependence (Figure 1, [63]). The minimum value of contractile force per cycle (end-diastolic) increases with increasing rate of stimulation as a result of the combination of factors: (a) increasing myoplasmic *Ca*^2+^ levels due to rate-dependent increase in SR release (Fig 6); and (b) decreasing time available for resequestering released *Ca*^2+^ from the myoplasm. The frequency dependence of the force response translates into a reciprocal rate dependent characteristic of the sarcomere length as shown in Figures 7E and F.

### Effect of maximal *β*-adrenergic stimulation

*β*-adrenergic stimulation bears a very crucial physiological relevance in normal functioning of the heart. We have investigated the effect of *β*-adrenergic stimulation on [ *Ca*^2+^] _*myo*_ under voltage clamp conditions. The degree of CaMKII mediated *I*_*Ca*,*L*_ facilitation is kept identical to the control case discussed earlier. Besides a moderate CaMKII-mediated [23,65] enhancement in *I*_*Ca*,*L*_, we observe that increase in stimulation frequency causes a strong *β*-adrenergic stimulation-dependent facilitation in *I*_*Ca*,*L*_, resulting in an enhanced positive slope in the FFR as the stimulation frequency is increased. These results tend to agree with studies that show that increased cAMP levels reverse the abnormalities in the FFR that are found in end-stage heart failure [66], and that moderate stimulation by isoproterenol partly reverses the negative FFR found in NYHA (New York Heart Association) class IV (severe) type heart failure and preserves the positive FFR in nonfailing myocardium [67]. It is important to note that variations in experimental protocol could result in undesirable artifacts owing to the time-sensitive nature of the effects of cAMP [68]. Here, we show that the presence of maximal *β*-adrenergic stimulation results in a stronger positive FFR (frequency dependence of peak contractile force generated) at physiological stimulation frequencies.

**Figure 8:**
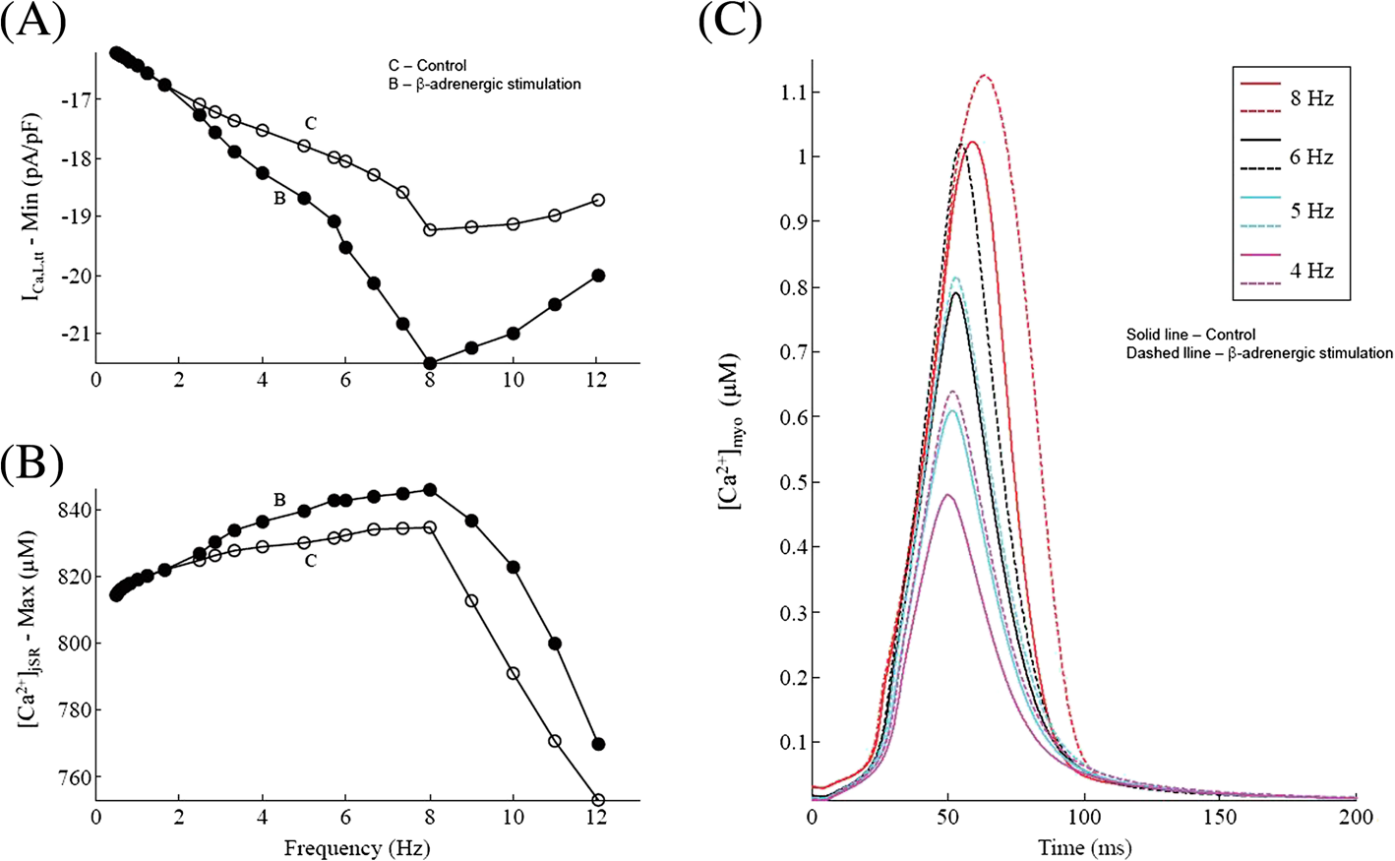
**Effect of maximal*β*-adrenergic stimulation on*Ca***^**2+**^** dynamics.** A comparison between cAMP-mediated maximal *β*-adrenergic stimulation and basal condition. Voltage clamp protocol used is a 50 ms step pulse to 10 mV from a holding potential of -40 mV. **(A)** Rate dependence of peak *I*_*Ca*,*L*_ current. **(B)** Frequency dependence of the maximum pre-release SR *Ca*^2+^ concentration. **(C)** [ *Ca*^2+^] _*myo*_ traces showing rate-dependent effects on systolic *Ca*^2+^ concentration in the myoplasm for increasing stimulation frequencies from 4.0 Hz to 8.0 Hz.

**Figure 9:**
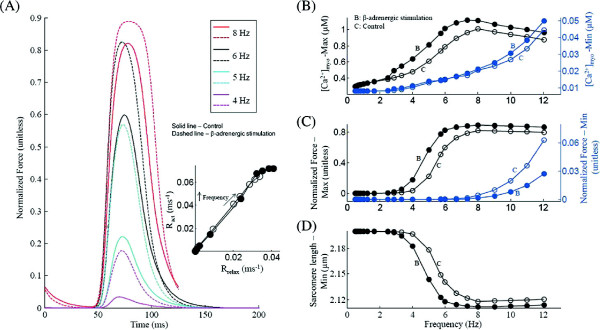
**Effect of maximal *β*-adrenergic stimulation on force response.** A comparison between cAMP-mediated maximal *β*-adrenergic stimulation and basal condition. Voltage clamp protocol used is a 50 ms step pulse to 10 mV from a holding potential of -40 mV. **(A)** Traces for normalized isometric force developed for increasing stimulation frequencies from 4.0 Hz to 8.0 Hz. The inset shows a plot of the maximum rate of relaxation (R _*relax*_) versus maximum rate of contraction (R _*act*_) for increasing stimulation frequency. **(B)** The frequency dependence of the maximum and minimum myoplasmic *Ca*^2+^ concentration. **(C)** Rate dependence of the maximum and minimum normalized force generated. **(D)** Frequency dependence of minimum sarcomere length achieved at peak contraction.

At low (< 2.5 Hz) stimulation frequencies *β*-adrenergic stimulation plays no role in altering intracellular *Ca*^2+^ dynamics. However, in the presence of maximal *β*-adrenergic stimulation (elevated levels of cAMP), an increase in stimulation rate (> 2.5 Hz) causes increased up-regulation of *I*_*Ca*,*L*_[69-71]. The model predicts (Figure 8A) that as the frequency of stimulation is increased in steps, peak *I*_*Ca*,*L*_ magnitude increases at a regular rate, before declining at high stimulation frequencies (> 8.0 Hz) as a result of incomplete channel recovery. As shown in Figure 8B, as the frequency is increased from 2.5 Hz to 8.0 Hz, the peak SR *Ca*^2+^ content increases due to two mechanisms, namely cAMP-dependent enhancement of both trigger current and a rate-dependent increase in uptake by the SERCA pump as a result of a stronger decline in the average level of unphosphorylated PLB, which stimulates the uptake. It is important to note that although the increase in peak [ *Ca*^2+^] _*myo*_ (traces in Figure 8C) causes a strong CaM-mediated CaMKII-dependent up-regulation of the SERCA pump due to an increase in PLB phosphorylation, cAMP-mediated phosphorylation of PLB causes further enhancement of the uptake mechanism. An increase in stimulation rate beyond 8.0 Hz, causes a decrease in SR *Ca*^2+^ content due to insufficient time for SR recovery along with a saturated operation of the SERCA pump. The presence of *β*-adrenergic stimulation significantly alters the relationship between the peak force response and the frequency of stimulation. As shown in the traces for force response in Figure 9A, besides an increase in peak force generated (a result of increased peak [ *Ca*^2+^] _*myo*_ (Figures 8C and 9B)), *β*-adrenergic stimulation causes an increase in contraction and relaxation rate. This cAMP-mediated increase in the rate of sarcomere contraction and relaxation is a combined result of increasing rate of rise and decline of the *Ca*^2+^-transient and a cAMP-mediated enhancement in rate kinetics governing cross-bridge formation. The inset in Figure 9A is a plot of the maximum rate of relaxation (R _*relax*_) versus maximum rate of contraction (R _*act*_) for increasing stimulation frequency with and without *β*-adrenergic stimulation (shows significant overlap). This linear relationship highlights contraction-relaxation coupling, and represents a key intrinsic property of the contractile myofilaments [72]. As shown in Figure 9B, although *β*-adrenergic stimulation further assists (due to the upregulation of *I*_*Ca*,*L*_ and SERCA pump) a CaMKII-mediated rate-dependent increase in peak [ *Ca*^2+^] _*myo*_, CaMKII is the key factor responsible for this behavior (a 2-fold increase in peak [ *Ca*^2+^] _*myo*_ from 4 Hz to 8 Hz both in the presence/absence of cAMP-mediated effects). In the presence of *β*-adrenergic stimulation, Figure 9C shows an enhancement in peak force generated and a decrease in minimum force as a result of a parallel trend in the peak systolic and end-diastolic levels of [ *Ca*^2+^] _*myo*_ (Figure 9B) supported by faster cross-bridge kinetics. The delta increment in force due to *β*-adrenergic stimulation (compared to the control case) is maximum at the stimulation rate of 5 Hz (Figure 9C) and decreases both above and below this frequency. The cAMP-mediated effect on the dependence of maximum sarcomere contraction (Figure 9D) on stimulation frequency mirrors that of the peak force response (Figure 9C) showing an opposite trend.

## Discussion

In this study, we have examined the role of two key mechanisms: (a) CaMKII-mediated upregulation; and (b) frequency-dependent cAMP-mediated *β*-adrenergic stimulation on *Ca*^2+^ cycling and their effect on the force-frequency response. In particular, we have modeled their two crucial signalling mechanisms: the rate-dependent upregulation of the DHP-sensitive *I*_*Ca*,*L*_ channel [25,69,73,74] and SERCA pump activity [75,76], which our study suggests are the key factors responsible for the characteristics of FFR in rat ventricular myocytes. These two signalling pathways mediate *I*_*Ca*,*L*_ enhancement bringing an extra supply of trigger current into the dyad to both enhance CICR and assist in a rate-dependent build up in pre-release diastolic SR *Ca*^2+^ concentration. This frequency-dependent increase in SR *Ca*^2+^ content is strongly assisted by a *β*-adrenergic stimulation-dependent cAMP-mediated increase in PLB phosphorylation, which relieves the inhibition exerted on the SERCA pump. This is especially important in the light of decreasing time for *Ca*^2+^ uptake with increasing stimulation rate.

We have systematically analyzed the important role of *Ca*^2+^-dependent CaM-mediated function of CaMKII and CaN as rate sensors. CaMKII is known to regulate EC coupling [41,45] and has substrates in both the dyadic domain (*I*_*Ca*,*L*_ and *I*_*ryr*_) [32,41] as well as the myoplasm (phospholamban) [77], and immunoprecipitates with the *I*_*Ca*,*L*_ channel [32] and RyR [78]. Our study confirms that activated-CaMKII causes rate-dependent acceleration of relaxation [3] both by direct phosphorylation of the SERCA pump as well as phosphorylation of PLB resulting in a decreased inhibition of the SERCA pump. Although the time course of the indirect effect of CaMKII via PLB phosphorylation is reported to be delayed with regard to frequency-dependent acceleration of relaxation [79], the direct phosphorylation of the SERCA pump by activated-CaMKII could cause an immediate increase in maximal pumping rate [4,23]. Despite poor sensitivity of CaMKII to myoplasmic *Ca*^2+^ signals due to its relatively low affinity for CaM and an inadequate supply of myoplasmic *Ca*^2+^-CaM complex (compared to the dyad), activated-CaMKII in the cytosol plays an important role in the enhancement of uptake via the SERCA pump except at high (> 8 Hz, inset in Figure 5A) stimulation frequencies. Here, the rapid decrease in time available for uptake results in gradually declining pre-release SR *Ca*^2+^ content. CaMKII has a more critical role in the dyadic cleft where we show [2] substantial CaMKII activity owing to large local *Ca*^2+^ concentration and CaM enrichment. It is reported that frequency-dependent CaMKII activity contributes to *I*_*Ca*,*L*_ channel facilitation [31] and assists a positive force-frequency relationship [80]. Our study shows that CaMKII causes a significant enhancement of *I*_*Ca*,*L*_ even under basal conditions (absence of *β*-adrenergic stimulation) as the stimulation frequency is increased to 8.0 Hz (Figure 4B), beyond which incomplete recovery begins to cause a decline in peak *I*_*Ca*,*L*_. A rate-dependent increase in SR *Ca*^2+^ content can be achieved only when the CaMKII-mediated (Figure 4C) increase in trigger current (Figure 4B) is supported by an increase in uptake via the SERCA pump by activated-CaMKII in the myoplasm (Figure 5C) which enhances PLB phosphorylation. CaMKII fails to sustain a positive FFR at very high (> 8.0 Hz) stimulation frequencies due to incomplete channel recovery causing a decline in trigger current and subsequently SR *Ca*^2+^ content. Data from atrial myocytes [81] show that myoplasmic CaN is also found to be responsive to increased pacing frequency.

Although CaN is also involved in *I*_*Ca*,*L*_ enhancement in the dyad at low (< 2.0 Hz) stimulation frequencies, at high stimulation rates (> 2.0 Hz), the large *Ca*^2+^ signals along with very slow dissociation of the *Ca*_4_*CaM*-CaN complex force it to be constitutively active, limiting its modulatory role. The presence of *β*-adrenergic stimulation, which also supports a sustained frequency-dependent increase in *I*_*Ca*,*L*_ (Figure 8A) and SERCA activity (Figure 8B) results in enhanced contractile response (Figure 9A and C).

Here, we have integrated an electro-mechanical model with our electrophysiological model of a rat ventricular myocyte [15] to describe isometric contractile force generated by myofibrils and investigate its rate-dependent characteristics. Our model for the cardiac contractile mechanism is derived from the description of cooperative activation and cross-bridge cycling given by Rice et al. [55]. However, we have incorporated a rate-dependent cAMP mediated enhancement in cross-bridge kinetics which is shown to be responsible for sustaining linear contraction-relaxation coupling (inset in Figure 9A) at increasing frequencies. It is important to note that the presence of CaMKII-mediated *I*_*Ca*,*L*_ enhancement or *β*-adrenergic stimulation results in physiologically relevant rate-dependent increase in peak cardiac contractile force (positive FFR).

### Model limitations

1. This model of a rat ventricular myocyte is limited to *Ca*^2+^ related channel, exchanger and pumps (*I*_*Ca*,*L*_, *I*_*NaCa*_, *I*_*PMCA*_, *I*_*ryr*_ and SERCA pump), while lacking exclusive *Na*^+^ or *K*^+^ related channels and transporters. Hence, it is aimed at mimicking voltage clamp conditions where channels other than calcium are blocked, and it cannot be used to study any action potential-induced *Ca*^2+^ transient-frequency relationship. However, its focus on the *Ca*^2+^ dynamics alone allows one to comprehend more clearly the important role of various *Ca*^2+^-dependent regulatory proteins such as CaMKII, CaN, PLB and cAMP in affecting multiple targets and thus generating a cell’s response to a change in the frequency of stimulation.

2. It is known that CaMKII alters the function of numerous ion channels and *Ca*^2+^ regulatory targets in a rate-dependent fashion. However, disparate findings exist on its modulation of targets such as the SERCA [82,83] and RyR channel [40,44,45]. Although our model aligns with rate-dependent CaN-mediated inhibition of the SERCA pump, its role in modulating SERCA activity is controversial due to conflicting findings [53,54]. Similarly, the effect of *β*-adrenergic stimulation via cAMP particularly, its dose-dependent influence on L-Type *Ca*^2+^ channels both in terms of modifying the single channel behavior such as *Ca*^2+^ ion permeability (channel conductance) as well as overall channel recruitment characteristics (open probability) is not clearly understood. While cAMP has been shown to increase channel open probability [35,36], increased levels of cAMP also result in increased phosphorylation of L-type *Ca*^2+^ channels, causing an increased permeability to *Ca*^2+^ ions [36-38]. Although we have modeled enhancement in *I*_*Ca*,*L*_ due to beta-adrenergic stimulation solely by a rate-dependent cAMP-mediated increase in channel conductance, the relative contribution of these factors to cAMP-dependent *I*_*Ca*,*L*_ enhancement has to be examined further. A detailed investigation is required to clarify the nature of these interactions. We hope that this study would help motivate more pointed experimental investigation of frequency-dependent CaMKII, CaN and cAMP effects on FFR in rat ventricular myocytes.

3. The effect of *β*-adrenergic stimulation on cardiac *Na*^+^/ *Ca*^2+^ exchange has been controversial. Perchenet et al. [84] report an enhancement in *Na*^+^/ *Ca*^2+^ exchange by the *β*-adrenergic/PKA-mediated phosphorylation of the exchanger protein. However, a cAMP-mediated enhancement in NCX activity would impede rate dependent increase in SR *Ca*^2+^ content. We base our model for the *Na*^+^/ *Ca*^2+^ exchanger on a more recent study by Lin et al. [85] which supports the view that *β*-adrenergic stimulation does not upregulate *Na*^+^/ *Ca*^2+^ exchange current.

4. The cooperative activation of the thin filament and the strain-dependent transitions of the cross-bridge cycle have been approximately modeled as non-spatial, state-variables. However, this simplification is valid as these transitions are inherently local phenomena and the model reproduces a wide range of steady state and dynamic responses in cardiac muscle [55].

## Conclusion

Using a mathematical model of an isolated rat ventricular myocyte in a voltage clamp setting, we have systematically examined the issue of rate-dependence in the proper functioning of the dyadic coupling unit, the regulation of SERCA function to provide adequate SR *Ca*^2+^ content, the peak amplitude of the myoplasmic *Ca*^2+^ transient and the complex interaction of all these factors. Given the complexity of these interacting systems, computer modeling gives an insight into the relative roles of different *Ca*^2+^ transport mechanisms. Our simulations explain the *Ca*^2+^-dependent, CaM-mediated, rate sensitive effects of CaMKII and CaN on various intracellular targets. We also investigate a significant, frequency-dependent, cAMP-mediated effect of *β*-adrenergic stimulation and its modulatory influence on the *I*_*Ca*,*L*_ channel as well as the SERCA pump. Rate-dependent CaMKII mediated *I*_*Ca*,*L*_ facilitation as well as cAMP-dependent upregulation of intracellular targets could play a vital role in reversing the negative FFR found in failing hearts. However, further studies are required to develop a clear understanding of the relative role of CaMKII and cAMP in the rate-dependent up-regulation of various intra-cellular targets especially the DHP-sensitive *I*_*Ca*,*L*_ channel. This would help in assigning rate-dependent weights to these signalling pathways. One could use KN-93 and autocamtide-2 related inhibitory peptide [86] for a study to delineate these effects. Our coupled electro-physiological and electro-mechanical model also sheds light on the rate dependence of the cardiac contractile mechanism. In particular, our model accounts for cAMP-dependent modulation of the rate kinetics governing cross-bridge formation. In agreement with Janssen [87], we also demonstrate a key linear relationship between the rate of contraction and relaxation, which is shown here to be intrinsically coupled over the full range of physiological frequencies both in the absence/presence of *β*-adrenergic stimulation. This study provides mechanistic, biophysically based explanations for the rate-dependent *Ca*^2+^ signalling underlying the force-frequency response in rat ventricular myocytes, generating useful and testable hypotheses.

## Appendix

Below is the set of equations modified in the model.

### Equations for currents modified (from Krishna et al. [15]) in the model

#### L-Type **C***a*^2+^ current

*Ca*^2+^ current through the DHP-sensitive *I*_*Ca*,*L*_ channel

(1) $$I_{\text{Ca},L}=I_{\text{Ca},L}^{§}\times F_{\text{cAMP},\text{Ical}}$$

(2) $$F_{\text{cAMP},\text{Ical}}=1.094-0.163\times \text{exp}(-0.219\times [\text{cAMP}])$$

(3) $$\xi^{‡}=550+6.0\times \text{CaMKI}I_{\text{act}}+\text{Ca}N_{\text{act}}$$

(4) $$\begin{array}{lll} \xi_{\text{new}}^{‡} & =550+\text{CaMKI}I_{\text{act}}\; & \;\\ & \;\times \left(1.348+\frac{\text{CaMKI}I_{\text{act}}^{3.22}}{3.135\times 10^{6}-0.755\times \text{CaMKI}I_{\text{act}}^{3.22}}\right)+\text{Ca}N_{\text{act}}\; \end{array}$$

*§*- *I*_*Ca*,*L*_ described in Krishna et al. [15]. *‡*- *ξ* described in Krishna et al. [15] causes rate-dependent modulation of the *I*_*Ca*,*L*_ channel.

#### Uptake of C*a*^2+^ from the cytosol into the LSR

Differential equation for phospholamban phosphorylation

(5) $$\begin{array}{l} \frac{\text{dPL}B_{\text{dp}}}{\text{dt}}=k_{12}^{\text{PLB}}(1+{\left(\text{Ca}N_{\text{act}}\times 10^{-4}\right)}^{2})\text{PL}B_{p}-k_{21}^{\text{PLB}}(1+{\left(\text{CaMKI}I_{\text{act}}\times 10^{5}\right)}^{2}\\ \;+F_{\text{cAMP},\text{SERCA}}^{2})\text{PL}B_{\text{dp}} \end{array}$$

(6) $$F_{\text{cAMP},\text{SERCA}}=0.1094-0.0163\times \text{exp}(-0.219\times [\text{cAMP}]\;)$$

(7) $$\text{PL}B_{p}=1-\text{PL}B_{\text{dp}}$$

$k_{12}^{\text{PLB}}$=6800 s ^−1^ ; $k_{21}^{\text{PLB}}$=1000 s ^−1^; (Krishna et al. [15]).

### Equations governing electro-mechanics modified (from Rice et al. [55]) in the model

Regulatory Ca ^2+^-binding to troponin

(8) $$\begin{array}{l} \frac{\text{dCaTro}p_{H}}{\text{dt}}=k_{\text{onT}}\text{Tn}I_{u}{\left[Ca^{2+}\right]}_{\text{myo}}(1-\text{CaTro}p_{H})\\ \;-k_{\text{off}\;\;\text{HT}}\text{CaTro}p_{H} \end{array}$$

(9) $$\begin{array}{l} \frac{\text{dCaTro}p_{L}}{\text{dt}}=k_{\text{onT}}\text{Tn}I_{u}{\left[Ca^{2+}\right]}_{\text{myo}}(1-\text{CaTro}p_{L})\\ \;-k_{\text{off}\;\;\text{LT}}\text{CaTro}p_{L} \end{array}$$

k _*onT*_ = 22.22 *μM*^−1^*s*^−1^ ; k _*offHT*_ = 17.36 s ^−1^; k _*offLT*_ = 173.61 s ^−1^; (Rice et al. [55]).

(10) $$\frac{\text{dTn}I_{p}}{\text{dt}}=k_{\text{onTI}}\Delta_{\text{PKA}}\text{Tn}I_{u}-k_{\text{off}\;\;\text{TI}}\text{Tn}I_{p}$$

k _*onTI*_ = 698.69 s ^−1^ ; k _*offTI*_ = 80.0 s ^−1^; (estimated from Roof et al. [88]).

(11) $$\text{Tn}I_{u}=1-\text{Tn}I_{p}$$

(12) $$\Delta_{\text{PKA}}=\frac{0.3\times [\text{cAMP}]}{[\text{cAMP}]+\;\;12.1}$$

(13) $$\text{fapp}T_{\text{new}}=\text{fapp}T^{‡}\times F_{\text{cAMP},\text{Force}}$$

(14) $$\;\text{hf}T_{\text{new}}=\text{hf}\;T^{‡}\times F_{\text{cAMP},\text{Force}}$$

(15) $$\;\text{hb}T_{\text{new}}=\text{hb}T^{‡}\times F_{\text{cAMP},\text{Force}}$$

(16) $$\;\text{gxb}T_{\text{new}}=\text{gxb}T^{‡}\times F_{\text{cAMP},\text{Force}}$$

(17) $$F_{\text{cAMP},\text{Force}}=1.873-1.4\times \text{exp}(-0.192\times [\text{cAMP}]\;)$$

‡- Rate constants governing cross-bridge kinetics described in Rice et al. [55].

## Abbreviations

[Ca2+]: calcium ion concentration; [Ca2+]jSR: luminal Ca2+ concentration in the jSR; [Ca2+]myo: myoplasmic Ca2+ concentration; [Ca2+]o: extracellular Ca2+ concentration; [Ca2+]ryr: Ca2+ concentration at the “mouth” of the RyR channel on the dyadic side; CaM: calmodulin; CaMKII: Ca2+/calmodulin-dependent protein kinase II; CaMKIIact: activated Ca2+/calmodulin-dependent protein kinase II; cAMP: cyclic adenosine monophosphate; CaN: calcineurin; CaNact: activated calcineurin; CDF: calcium-dependent facilitation; CDI: calcium-dependent inactivation; CICR: calcium-induced calcium-release; CRCP: contraction-relaxation coupling point; DCU: dyadic coupling unit; DHP: dihydropyridine; DHPR: dihydropyridine receptor; E-C: excitation contraction; EC50bwd: affinity of backward Ca2+ flux from LSR to myoplasm; : ; ICa,L: L-type Ca2+ current; Icyt,serca: Ca2+ uptake current directed from the myoplasm to the SERCA; INa,b: background sodium current; INaCa: sodium calcium exchanger current; INaCs: sodium cesium pump current; IPMCA: plasma membrane Ca2+ ATPase pump current; Iryr: Ca2+ current due to CICR from an individual jSR; Iserca,sr: Ca2+ uptake current directed from the SERCA to the LSR; jSR: junctional portion of the sarcoplasmic reticulum; LCC: L-type DHP-sensitive Ca2+ channel; L-type: long lasting type; LSR: longitudinal portion of the sarcoplasmic reticulum; mM: milli molar; mV: milli volt; [Na+]myo: myoplasmic Na+ concentration; NCX: Na+/Ca2+ exchanger; nM: nano molar; NO: Nitric oxide; pC: pico coulomb; Po: Open probability; PKA: protein kinase A; PLB: phospholamban; : ; PLBp: phosphorylated phospholamban; PSR: phospholamban to SERCA ratio; Ry: ryanodine; RyR: ryanodine receptor; SERCA: sarcoplasmic reticulum Ca2+ ATPase; SL: sarcolemma; SR: sarcoplasmic reticulum; VC: voltage clamp; VDI: voltage-dependent inactivation.

## Competing interests

The authors declare that they have no competing interests.

## Authors’ contributions

AK developed the coupled electromechanical model of the rat ventricular myocyte, carried out the voltage clamp modeling studies, and drafted the manuscript. MV made substantial intellectual contributions to the study and in drafting of the manuscript. PTP made intellectual contributions to the manuscript as well as significant contributions to the drafting of the manuscript. JWC made key contributions to the conception of the study, design, analysis and interpretation of results, and drafting of the manuscript. All authors read and approved the final manuscript.
